# ﻿Editorial

**DOI:** 10.3897/phytokeys.205.89634

**Published:** 2022-08-22

**Authors:** Colin E. Hughes, Luciano P. de Queiroz, Gwilym P. Lewis

**Affiliations:** 1 Department of Systematic & Evolutionary Botany, University of Zurich, Zollikerstrasse 107, 8008 Zurich, Switzerland University of Zurich Zurich Switzerland; 2 Universidade Estadual de Feira de Santana, Departamento de Cièncias Biológicas, Av. Transnordestina s.n., Novo Horizonte, Feira de Santana, BA, 44036-900, Brazil Universidade Estadual de Feira de Santana Feira de Santana Brazil; 3 Accelerated Taxonomy Department, Royal Botanic Gardens, Kew, Richmond, Surrey, TW9 3AE, UK Accelerated Taxonomy Department, Royal Botanic Gardens Richmond United Kingdom

Since its inception in 1981, the Advances in Legume Systematics (ALS) series has provided an important outlet for publishing new results on all aspects of the systematics and classification of legumes, including papers arising from the seven International Legume Conferences. The first ten parts in the ALS series were published by the Royal Botanic Gardens, Kew, and subsequent parts as Special Issues of two botanical journals – “Australian Systematic Botany” (Part 11 in 2003 and Part 13 in 2019) and “South African Journal of Botany” (Part 12 in 2013) – a format continued here for Part 14 with this Special Issue of “PhytoKeys”.

Here in ALS 14 the focus is on classification of the legume subfamily Caesalpinioideae, as it was re-circumscribed by the Legume Phylogeny Working Group (LPWG) in 2017. Caesalpinioideae is the second largest subfamily of legumes with ca. 4,600 species currently placed in 152 genera. Within the subfamily, ca. 3,400 species and 90 genera are placed in the mimosoid clade corresponding to the former subfamily Mimosoideae, which is nested within new sense Caesalpinioideae. The subfamily has a pantropical distribution and many of its lineages form diverse and ecologically abundant or dominant elements across dry, savanna and wet lowland tropical biomes. Despite major advances in the last few decades towards aligning genera with clades, generic delimitation in Caesalpinioideae remains in a state of considerable flux, especially across the mimosoid clade.

In the introductory paper of this Special Issue, a new phylogenomic framework for Caesalpinioideae built from DNA sequences of 997 nuclear genes for 420 species of 147 of the 152 genera recognized in the subfamily prior to ALS14, is presented. This new phylogeny reveals that 22 genera are non-monophyletic or nested within another genus and underpins a series of 15 papers focused on generic delimitation of particular subclades, which are presented here in ALS14 Part 1. This phylogeny also provides the framework for a new higher-level tribal and clade-based classification including a synopsis of genera in the subfamily which will be presented separately in ALS14 Part 2.

Here in ALS14 Part 1, the 16 papers are authored by 52 authors from 13 countries. Nine new genera are described, five genera are reinstated and three genera are subsumed into synonymy in other genera. With addition of these 14 new and reinstated genera and subtraction of three genera placed in synonymy, the total number of genera in subfamily Caesalpinioideae now stands at 163, of which 102 are in the mimosoid clade. One new species is described, several new sections of genera are erected, and 139 new nomenclatural combinations are proposed.

Given the extent of the adjustments to generic limits presented here in ALS14 Part 1, it is clear that this focus on generic delimitation was much needed. Of the 22 instances of generic non-monophyly, 15 have been reclassified here in ALS14 representing a significant step towards aligning genera with clades across Caesalpinioideae. A fully updated synopsis of the now 163 genera forms part of the new classification of Caesalpinioideae presented in ALS14 Part 2.

As editors, we thank the many people and organisations who have helped to bring this ALS14 Special Issue to fruition. The foundations for ALS14 were established through phylogenomic work started by Erik Koenen, and it was his idea to use the results of these new phylogenomic analyses to assemble a compilation of papers focused on generic delimitation authored by many different people in a single volume as part of the ALS series. We thank Erik for his vision and many contributions to making that happen. We are extremely grateful to Patrick Herendeen who acted as an additional handling editor for the introductory phylogenomics paper on which all three of us as editors are authors. We thank all the people who have promptly and willingly reviewed papers: Stephen Boatwright, Leonardo Borges, Gillian Brown, Anne Bruneau, Warren Cardinal-McTeague, Domingos Cardoso, Else Demeulenaere, Rafael Govaerts, Ethiéne Guerra, William Hawthorne, Héctor Hernández, Stefanie Ickert-Bond, Erik Koenen, Melissa Luckow, Marli Morim, Dan Murphy, Toby Pennington, Marianne Le Roux, Marcelo Simon and Jan Wieringa, and Dóra Huszár for help to compile the Index. We also very much appreciate the hard work of the Pensoft editorial team and especially the managing editor of PhytoKeys, Yasen Mutafchiev who has overseen production of this Special Issue. Finally, we thank Swiss National Science Foundation (grant 31003A_182453/1 to CEH) for support that underpinned research presented in this Special Issue, and the following organisations for contributing funds to support Open Access publication costs of ALS14 Part 1: Royal Botanic Gardens, Kew, U.K. (O’Donnell et al. and Clark et al. papers), the U.S. National Science Foundation grant number OIA-1946352 to the University of Guam (Demeulenaere et al. paper), the Australia and Pacific Science Foundation, Australia (Brown et al. paper), and especially the Department of Systematic and Evolutionary Botany at the University of Zurich, Switzerland (all other papers).

